# Integration of Remote Sensing and Social Sensing Data in a Deep Learning Framework for Hourly Urban PM_2.5_ Mapping

**DOI:** 10.3390/ijerph16214102

**Published:** 2019-10-24

**Authors:** Huanfeng Shen, Man Zhou, Tongwen Li, Chao Zeng

**Affiliations:** 1 School of Resource and Environmental Sciences, Wuhan University, Wuhan 430079, China; shenhf@whu.edu.cn (H.S.); ZhouM@whu.edu.cn (M.Z.); litw@whu.edu.cn (T.L.); 2 Collaborative Innovation Center of Geospatial Technology, Wuhan 430079, China; 3 The Key Laboratory of Geographic Information System, Ministry of Education, Wuhan University, Wuhan 430079, China

**Keywords:** PM_2.5_, social sensing, remote sensing, feature extraction, deep learning

## Abstract

Fine spatiotemporal mapping of PM_2.5_ concentration in urban areas is of great significance in epidemiologic research. However, both the diversity and the complex nonlinear relationships of PM_2.5_ influencing factors pose challenges for accurate mapping. To address these issues, we innovatively combined social sensing data with remote sensing data and other auxiliary variables, which can bring both natural and social factors into the modeling; meanwhile, we used a deep learning method to learn the nonlinear relationships. The geospatial analysis methods were applied to realize effective feature extraction of the social sensing data and a grid matching process was carried out to integrate the spatiotemporal multi-source heterogeneous data. Based on this research strategy, we finally generated hourly PM_2.5_ concentration data at a spatial resolution of 0.01°. This method was successfully applied to the central urban area of Wuhan in China, which the optimal result of the 10-fold cross-validation *R*^2^ was 0.832. Our work indicated that the real-time check-in and traffic index variables can improve both quantitative and mapping results. The mapping results could be potentially applied for urban environmental monitoring, pollution exposure assessment, and health risk research.

## 1. Introduction

Fine particles with an aerodynamic diameter of less than 2.5 micrometers (PM_2.5_), which correspond to the “high-risk respirable convention”, as defined in [1], have aroused worldwide concern [2]. The troubling thing is that 92% of the world population are exposed to PM_2.5_ air pollution concentration that is above the annual mean World Health Organization Air Quality Guidelines (WHO AQG) level of 10 μg/m^3^ [3]. In addition to the health effects, PM_2.5_ also has significant impacts on climate change, agricultural production and ecological environment [4].

To our knowledge, a number of studies have explored the effects of PM_2.5_ on health and evaluated population exposure to PM_2.5_, based on a continuous distribution of PM_2.5_ [5,6]. And the results showed that the accuracy of the PM_2.5_ concentration estimation has a great impact on the research conclusions, and the spatiotemporal PM_2.5_ distribution data are very important basic data. However, ground monitoring stations for PM_2.5_ are limited up to now, because these static and expensive facilities are often sparsely and unevenly distributed in study area [7]. Hence, generating accurate fine spatiotemporal mapping of PM_2.5_ concentration is important to meet the practical demand.

There are two main widely used types of methods for obtaining a continuous distribution of PM_2.5_, one of which is simulation models that are based on scientific cognition of the physical and chemical processes of the atmosphere [8,9], and the other is statistical models [10,11,12,13,14,15,16,17,18,19,20,21,22,23,24]. The simulation models require model parameters, drivers and initial conditions, which lead to much uncertainty and computational complexity. While the statistical models, especially the machine learning methods are easier to implement as they do not consider the complex physicochemical processes. In the existing research on the application of statistical methods to estimate air pollutants, the more typical methods include: the Land Use Regression method (LUR) [15,22,23,24] and the Mixed Linear Model (LME) [20,21], the Geographically Weighted Regression model (GWR) [10,19], which are widely used in atmospheric pollutants estimation and spatiotemporal analysis, but their ability to describe nonlinear relationships between variables is limited; some machine learning methods, such as the Artificial Neural Networks (ANN) [11,15], Generalized Regression Neural Networks (GRNN) [25], Random Forests (RF) [13,16,18], eXtreme Gradient Boosting (XGBoost) [16] and Bagging Regression [21], etc. These models show higher precision and stability. Especially the more advanced deep learning methods [14,26,27] have stronger learning ability for complex nonlinear relations. In addition, some methods [11,15,19,21] proposed the integrated learning methods and step-by-step strategies to apply the statistical models, which could improve the accuracy. Nowadays the increasing number of ground monitoring stations around the world can provide abundant modeling samples that can be applied to the statistical models, thus more reliable and accurate estimation results can be obtained. Moreover, the rapid development of both remote sensing and social sensing technologies can provide us broad views for spatiotemporal estimation of air pollution based on advanced statistical methods [28,29,30].

On the one hand, remote sensing data are widely used in PM_2.5_ estimation studies, especially the aerosol optical thickness (AOT) products, which have been proved to be closely related to PM_2.5_ concentration [25,26,31]. Moderate Resolution Imaging Spectroradiometer (MODIS) AOT products provide a broad basis for PM_2.5_ estimation [10,15,16,32]. In recent years, geostationary satellites have been successfully launched and operated, which have the advantages of short revisit periods compared with the polar orbit satellites. Examples of such satellites are the Geostationary Ocean Color Imager (GOCI), Fengyun-4 and Himawari-8. Zhang [20] and Wang [21] derived hourly particulate matter concentration from the Himawari-8 AOT product, which are meaningful attempts at introducing high-resolution remote sensing data into the hourly mapping of PM_2.5_.

On the other hand, some studies have made full use of anonymized and passively collected social sensing data to estimate air pollutants. Social sensing data, such as point of interest (POI) data, check-in data, floating car data, and so on, can give a deep insight into human society [33]. As early as 2013, Zheng [11] inferred an urban air quality index with multiple big data (including vehicle trajectory data, POI data, road network data, and meteorological features), using a spatial and temporal co-training method. The innovative method was successfully applied in case studies of Beijing and Shanghai and provided a good foundation for the later research. Zhu [12] also used urban big data (including meteorological data, traffic index data, vehicle velocity data, road saturation data, POI data, and urban form data) for air quality estimation in Shenzhen and Hong Kong, and Lin [13] utilized publicly available Open Street Map (OSM) data to generate PM_2.5_ in the Los Angeles metropolitan area. These studies have confirmed the potential of social sensing data for inferring air quality.

To date, few studies have integrated remote sensing data and social sensing data for the spatiotemporal mapping of PM_2.5_. Considering that remote sensors are still incapable to record socioeconomic and human activity attributes, and that social sensing data insufficiently encompass natural factors, taking advantage of both the reliability of remote sensing data and the spatiotemporal dynamic characteristic of social sensing data is a promising research direction [28]. Xu [16] combined Feng Yun satellite AOT data with POI data, road network data and meteorological data, using a two-stage inference approach to infer a daily air quality index (AQI) in Beijing, which performed better than the method without remote sensing data [11]; Brokamp [18] and Xiao [19] also tried to combine MODIS AOT data with some static social data, including road network data and population data, for estimating daily and monthly PM_2.5_, respectively. However, these studies only attempted to use relatively static social sensing variables for estimating PM_2.5_ at a daily or monthly scale, and the modeling approaches were relatively simple for mining complex relationships among multi-variables.

It is worth noting that there are many factors that are related to PM_2.5_, including meteorological influences, atmospheric boundary layer height, land use types, urban form, traffic conditions, human activities, and so on [34,35,36,37,38,39]. To mine the complex relationships between the various influencing factors and PM_2.5_, machine learning methods [11,13,14,15,16,17,18,19] have been widely used, especially the deep learning methods [27,40]. The traditional methods cannot explain complex nonlinear relationships well, whereas the deep learning models can extract effective features from multi-variable and complex relationships, due to their strong learning ability. To the best of our knowledge, no studies, to date, have used a deep learning framework to combine both remote sensing data and social sensing data for the spatiotemporal mapping of PM_2.5_. It is considerable to make full use of deep learning method to mine the “big data” [30].

In the study, we focused on overcoming the challenges caused by the diversity and the complex nonlinear relationships of PM_2.5_ influencing factors to the accurate PM_2.5_ estimation of fine spatiotemporal resolution. We introduced dynamic social sensing data integrated with remote sensing products using a spatiotemporal grid-matching model framework and utilized a deep belief network for multi-variable mining. This method was applied to the central urban area of Wuhan in China to generate an hourly 0.01° PM_2.5_ mapping result. Finally, we discussed the effects of variables.

## 2. Study Area and Data

### 2.1. Study Area

**Figure 1:**
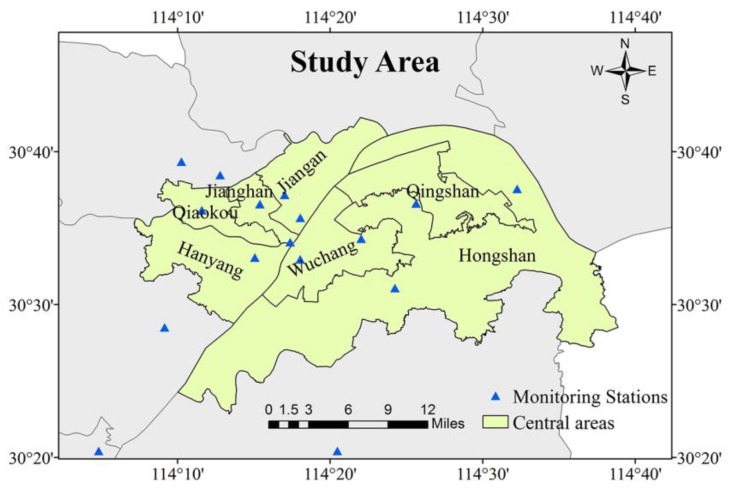
Study region and the distribution of ground monitoring stations.

The study region shown in Figure 1 is the central urban area of Wuhan, which is the largest city in central China. According to the statistics [4], the PM_2.5_ concentration level in Wuhan is above the average level in China. In Wuhan, approximately 60% of the permanent population lives on about 10% of the land in the central urban area [41], so that we need to pay attention to the effect of PM_2.5_ pollution on human and environmental health [42]. Meanwhile, Wuhan is the core city of the Yangtze River Economic Belt, and also a comprehensive transportation hub for China. Thus, the ecological construction of Wuhan is particularly important, and the spatiotemporally continuous mapping of PM_2.5_ is essential for environment monitoring. Given all of the above, we chose this area as a case study, as the existing studies have rarely considered this region.

### 2.2. Data Sets

In this study, we took five categories of data (from 24 January 2018 to 31 July 2018) into consideration: ground station PM_2.5_ data, social sensing data, remote sensing data, meteorological data and terrain data.

#### 2.2.1. Ground Station PM_2.5_

Hourly PM_2.5_ data for the study period were obtained from the China National Environmental Monitoring Center (CNEMC) web site (http://www.cnemc.cn/) and the Hubei Environmental Monitoring Center (http://hbt.hubei.gov.cn/hjxx/). There are 11 ground monitoring stations in the central area of Wuhan. We also took the neighboring sites into consideration in the modeling process, so that 20 stations, in total, were considered in our experiment. About 90,000 PM_2.5_ concentration records were collected for the study period.

#### 2.2.2. Social Sensing Data Remote Sensing Data

We collected four kinds of social sensing data: real-time check-in data, traffic index data, road network data and POIs. The first two are dynamic real-time data, and the last two are relatively static data.

Firstly, real-time check-in data were obtained from the “Tencent Location Big Data” service (https://heat.qq.com/). This service updates every 5 min with a spatial resolution of approximately 0.01° for about 6000 location points in Wuhan. The anonymized and passively collected geolocation data allow the analysis of population activity and mobile patterns.

Secondly, traffic index data were gathered from the NavInfo Traffic Index platform (http://www.nitrafficindex.com/). The traffic index is a quantitative indicator which has six levels on the basis of the actual road speed and road conditions, and also the subjective feeling degree about traffic congestion of people is added to describe the road traffic operation status. We obtained the traffic index data of 502 Roads in Wuhan in each hour which can be used as a factor to reflect the real-time traffic influence on the atmosphere.

Thirdly, road network data were downloaded from OSM (https://www.openstreetmap.org/), which was updated in 2018.

Finally, POIs were obtained from the Amap developer platform (https://lbs.amap.com/), including company enterprises, traffic facilities, road facilities, scenic spots, and other types.

#### 2.2.3. Remote Sensing Data

Two kinds of products were used in our study, one closely corresponding to the ground station PM_2.5_ and the other reflecting the land-cover information. We chose the Himawari-8 Level 3 hourly AOT product based on the method developed by Yoshida*,* et al. [43] and subsequently improved by Kikuchi, et al. [44], with strict cloud screening using the differences in the spatiotemporal variability characteristic of aerosol and cloud. This product has a good quality but a poor spatial coverage. Hourly AOT data for the study period with a spatial resolution of 5-km were downloaded from the Japan Aerospace Exploration Agency (JAXA) P-Tree System (http://www.eorc.jaxa.jp/ptree/). In this study, only aerosol retrievals with the highest confidence level (“very good”) were adopted for the estimation of PM_2.5_. The MODIS normalized difference vegetation index (NDVI) was used for the presentation of land use types. The 16-day synthetic product with a spatial resolution of 1-km (MOD13Q1) was downloaded from the Level-1 and Atmosphere Archive & Distribution System (LAADS) Web site (http://ladsweb.nascom.nasa.gov/).

#### 2.2.4. Meteorological Data

Hourly specific relative humidity (RH, %), air temperature at a 2-m height (TEM, *K*), east wind speed and north wind speed at 10-m above ground (EWS, NWS, m/s), surface pressure (SP, kpa), and planetary boundary layer height (PBLH, m) data were obtained from the Goddard Earth Observing System Data Assimilation System (GEOS 5-FP) (https://fluid.nccs.nasa.gov/weather/). These reanalysis meteorological data have a spatial resolution of 0.25° latitude × 0.3125° longitude.

#### 2.2.5. Terrain Data

We used the NASA Shuttle Radar Topographic Mission (SRTM) digital elevation model (DEM) product as terrain data, which has a resolution of 90 m at the equator. The data were obtained from the Consultative Group on International Agricultural Research-Consortium for Spatial Information (CGIAR-CSI) (http://srtm.csi.cgiar.org/).

## 3. Methods

**Figure 2:**
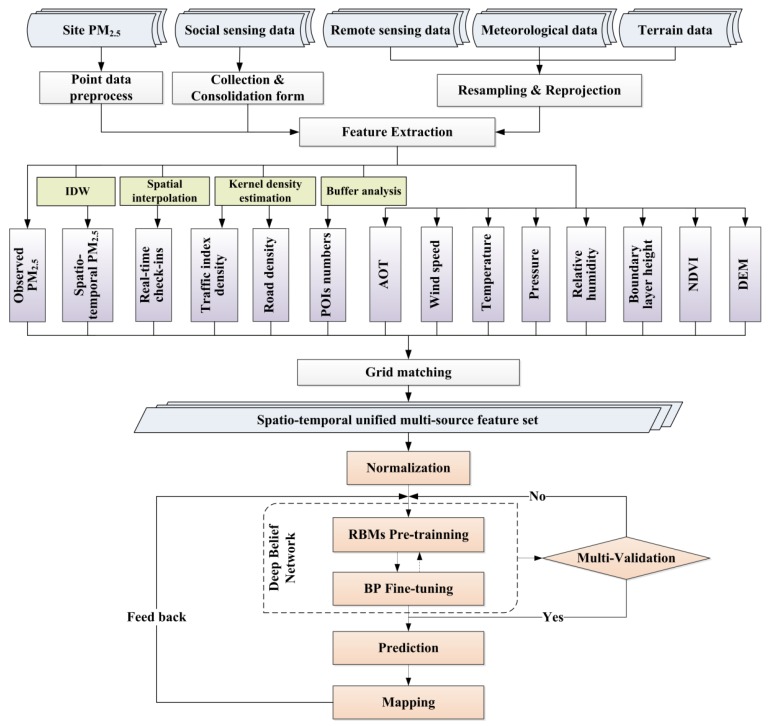
Data and modeling processes. “IDW” means inverse distance weighting method; “POI” means points of interest; “AOT” means aerosol optical thickness product; “NDVI” means normalized difference vegetation index product; “DEM” means digital elevation model data; “RBM” means restricted Boltzmann machine; “BP” means the back-propagation neural network.

Figure 2 shows the data and modeling processes. We performed data preprocessing on the original multi-data, then employed geospatial analysis methods and image processing means to construct and extract the input variables (The abbreviations for data sets and variables are summarized in Supplementary Material: Table S1). When all the variables were converted to the raster format, we matched the grids of multiple variables on a specific hour scale. Then the multivariate vector of each labeled grid (including the site-based PM_2.5_ observation) could be obtained, which is also the form of the model input sample. Finally, a spatiotemporally uniform multi-source feature set could be used for modeling. We used the deep belief network (DBN) model [45] in the study, which is one of the most classic deep learning models. The quantitative evaluation and mapping feedback were both considered for obtaining more reliable and accurate results. The feature extraction and DBN model are explained in detail in the following.

### 3.1. Feature Extraction

While there are many ways to construct and extract the input variables, some of them fairly involved, we attempted to use geospatial analysis, geostatistics, and image processing methods to extract features from multi-source heterogeneous data effectively and easily. Then the features with different spatiotemporal scales and different data formats were unified into the raster format on the same scale, which could be easily used to generate sample data for modeling.

#### 3.1.1. Spatiotemporal Features of PM_2.5_

**Figure 3:**
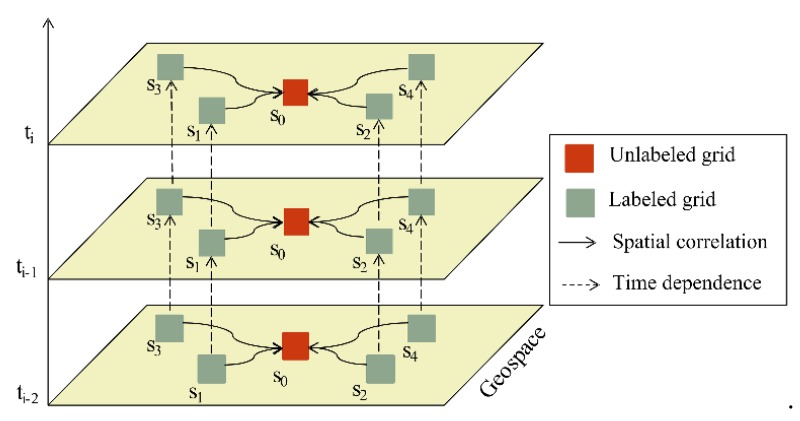
Schematic diagram of calculating the spatiotemporal features of PM_2.5_. The t_i_, t_i-1_, t_i-2_ represent one hour, two hours, and three hours before the current moment respectively. The labeled grids (s_1_, s_2_, s_3_, s_4_) represent the places that include ground monitoring stations, while the unlabeled grid (s_0_) represents the space that needs to be estimated.

The spatiotemporal distribution of PM_2.5_ concentration follows Tobler’s First Law of Geography [46], which means that near things are more related to each other. According to the spatiotemporal autocorrelation of PM_2.5_, we calculated the characteristic variables of the initial distribution of concentration. Figure 3 shows the spatial correlation and time dependence of the unlabeled grid which can be inferred by the adjacent labeled grids that have observed PM_2.5_ concentrations. The inverse distance weighting (IDW) method was used to calculate the spatial feature of PM_2.5_ (PM_s_) and the temporal feature of PM_2.5_ (PM_t_).

#### 3.1.2. Social Sensing Features

(1) Real-Time Check-In

Compared with traditional demographic data, Tencent real-time check-in data can dynamically reflect the spatial distribution and temporal variation characteristics of population. The high correlation between the check-in density of social media data and human density distribution has been revealed by many studies [47,48,49]. The raw data are in JavaScript Object Notation (JSON) format. We transcoded and vectorized the data to get the point data with a 0.01° spatial resolution. Considering that the instability and uncertainty of the acquisition of social sensing data would cause the absence of data in some regions or at some points, we used the IDW spatial interpolation method to fill the gaps and also get the raster data. The numbers of check-ins of each grid represent the distribution of population. Finally, the hourly averaged real-time check-in (RTCI) feature was adopted in our model.

(2) Traffic Index Density

Real-time traffic index data can reflect traffic flow information. Studies such as Forehead and Huynh [38] have proved that automobile exhaust emissions have a great impact on PM_2.5_ pollution, where PM_2.5_ concentration often rises in times of traffic congestion or at rush hour. The raw data were converted to the line features with traffic index attribute. Then the kernel density analysis (KDA) method [50] was used to estimate the kernel density of the traffic index in study area, so as to obtain the hourly raster data of the traffic index density feature (TID) at a 0.01° spatial resolution. The KDA could capture the spot of traffic congestion and could quickly calculate the spatial distribution of the line features.

(3) Road Network Density

The road network can reflect the spatial pattern of a city. Its form and layout often divide an urban system into blocks of different sizes and different functional areas. Four levels of roads were concluded in this study, i.e., highways, main roads, secondary roads and branch roads. The ratio of the total length of the roads to a certain area was regarded as the density of the road network (ROAD). ROAD was a static variable during study period.

(4) POIs

POIs can be regarded as a mass of points of interest abstracted from various entities in a city, including infrastructures, business districts, catering and entertainment places, office buildings, industrial enterprises, scenic spots, etc. POIs are a portrait of the whole city and reflect the appearance of urban development. Allowing for the fact that not all POIs and PM_2.5_ are relevant, we filtered two groups of representative characteristic variables from all the POIs: the potential sources of pollution type (PS), including chemical plants, steel mills, textile factories, printworks, and others; and the cleaner location type (Scen), including water bodies, parks, and scenic spots. The buffer analysis method was used to calculate the POI numbers within the buffer range. Finally, each grid was given the value of the number of each type of POIs.

#### 3.1.3. Other Raster Features

The remote sensing data (AOT and NDVI), meteorological data, and DEM data are all organized as raster data originally. We resampled the raster data at a 0.01° spatial resolution. For the NDVI product with a revisit period of 16 days, multi-scene data sets corresponding to each 16-day period during the research period were generated and successively arranged; and the DEM data remained unchanged during the study period. Finally, we obtained features of AOT, NDVI, DEM, RH, TEM, EWS, NWS, SP, and PBLH.

### 3.2. Deep Learning Model for PM_2.5_ Estimation

**Figure 4:**
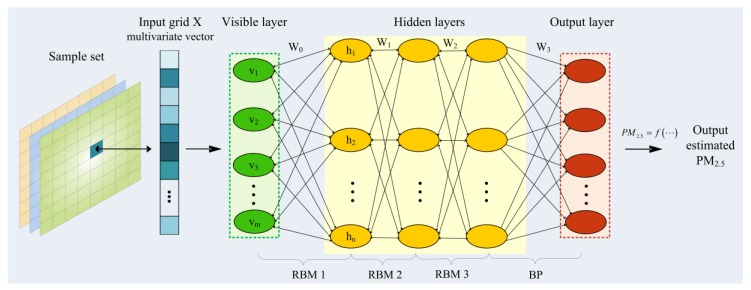
The structure of the DBN for the estimation of PM_2.5_. “DBN” means deep belief network.

Deep learning is able to mine complex and nonlinear relationships between many variables, so as to provide the prospect that effectively predicts the spatial and temporal distribution of PM_2.5_. We used the DBN model that outperforms the other traditional algorithms on PM_2.5_ estimation [27]. It is an alternatively a class of simple, unsupervised networks such as restricted Boltzmann machines (RBMs), composed of multiple layers of latent variables, with connections between the layers but not between units within each layer [45]. As shown in Figure 4, our training model has 3 RBM layers and selects a back-propagation (BP) neural network as the prediction method. The input is labeled samples that each sample involves the ground truth monitoring values cooperating with the multi-source variables, shown that each grid X corresponds to a multivariate vector. In order to eliminate dimensionality and accelerate the convergence speed of this model, the Min-Max Normalization method was adopted before training. The output layer contains the learned weights of each neuron. Finally, the general structure used to estimate PM_2.5_ is:

(1) $${PM}_{2.5}=f(Time,SSD,RSD,PM_{s},PM_{t},Wea,DEM)$$

where *SSD* means the social sensing variables, including *RTCI*, *TID*, *POIs*, and *ROAD*; and *RSD* includes *AOT* and *NDVI*; and the meteorological variable *Wea* contains *WS*, *RH*, *PBLH*, *TEMP*, and *SP*. All the input variables were explained earlier in Section 3.1. The three main parts of the model process are as follows.

(1) Pre-Training

The pre-training process was performed by a series of RBM layers, as shown in Figure 4, which is a method of generating model weights by unsupervised learning from layer to layer. One RBM is a two-layer network, including a visible layer (*v*) of m neurons and a hidden layer (*h*) of n neurons, both of which are connected by weights (W) [51]. A training method called contrastive divergence (CD) [52] was used to get the weight updated. The activation probability of each neuron in the hidden layer was calculated as shown in (2). Similarly, the conditional distribution probability of reconstructing the visible layer with the hidden layer was calculated as shown in (3).

(2) $$p(h_{j}=1/v)=\frac{p(h_{j}=1,v)}{p(v)}=\log sig(\sum_{i=1}^{m}w_{ij}v_{i}+c_{j})$$

(3) $$p(v_{i}=1/h)=\frac{p(v_{i}=1,h)}{p(h)}=\log sig(\sum_{j=1}^{n}w_{ij}h_{j}+b_{i})$$

where $b_{i}$ and $c_{j}$ are the bias of the ith visible neuron $v_{i}$ and the jth hidden neuron $h_{j}$, respectively; $w_{ij}$ is the weight between the two neurons. The logsig indicates the activation function $\log sig(x)=\frac{1}{1+\exp(-x)}$, which introduced the nonlinear characteristics into our network.

By calculating the activation probability of the hidden layer neuron inferred from the real visible layer as $p(h_{j}|v_{idata})$ and that inferred from the visible layer reconstructed from the hidden layer as $p(h_{j}|v_{ireconstruction})$, the weights and bias parameters were updated as

(4) $$\begin{array}{l} w_{ij}\leftarrow w_{ij}+\lambda (p(h_{j}|v_{idata})v_{idata}-p(h_{j}|v_{ireconstruct})v_{ireconstruct})\\ b_{i}\leftarrow b_{i}+\lambda (v_{idata}-v_{ireconstruct})\\ c_{j}\leftarrow c_{j}+\lambda (p(h_{j}|v_{idata})-p(h_{j}|v_{ireconstruct})) \end{array}$$

where λ is the learning rate. After the CD training process, the hidden layer can not only accurately express the characteristics of the input features of the visible layer, but it can also reconstruct the visible layer. We carried out many experiments to adjust the optimum network parameters, and finally chose 3 hidden layers, with the neuron number of each layer being 12, 24 and 36, so that the multi-layer RBM could realize deep feature extraction.

(2) Fine-Tuning

We selected the BP neural network to achieve the fine-tuning of the entire network, which can reverse the PM_2.5_ estimation error to each RBM, layer by layer. The mean-squared normalized error performance function (MSE) was used to measure the network error as

(5) $$E=\frac{1}{n}\sum_{p=1}^{n}(y(p)-\overset{\wedge }{y}(p){)}^{2}$$

where n is the total number of samples; and y(p),$\overset{\wedge }{y}$(p) are the target value and the output value of the $p_{th}$ input sample respectively. Whether to reverse the error information or not depends on whether the condition is satisfied. When the error meets the preset accuracy or the number of iterations reaches the upper limit, the algorithm is terminated.

In the BP network, the weight of the output of the RBMs was used as the input, which overcame the shortcoming of the BP falling into local optima due to the random initialization of the weight parameters. The weight of each layer was updated by

(6) w←w−η∇E(w)

where η is the learning rate, and ∇E(w) is the partial derivative of the network error, with respect to the weight of a certain layer. We used the Levenberg-Marquardt (L-M) backpropagation method [53] as the training function for achieving the optimal solution of the minimized error. The L-M can accelerate the speed of convergence and avoid getting trapped in local optima.

(3) Prediction

This step was based on the reiterative validations of the DBN model. We selected the most appropriate network parameters in the training process with the labeled grid samples. The trained DBN network *net* and the setting of the normalized parameters were saved for the prediction as

(7) $$\mathrm{Pr}e\_PM_{2.5}=sim(net,X_{input\_reg})$$

where $X_{input\_reg}$ is the unlabeled grid vectors, which were normalized using the same normalized parameters as the training input data. Thus, the spatially continuous PM_2.5_ concentration can be obtained by using the simulation function sim.

### 3.3. Validation

We carried out both the quantitative verification and the mapping test to obtain more reliable network parameters and more accurate estimation results. A 10-fold cross-validation (CV) method [54] was used to evaluate the overall estimation capability of the DBN model. Specifically, the sample set was randomly divided into 10 parts, with one part as the validation set and the others as the training set. The 10 data parts were then successively verified and, finally, the average value of the results of the 10 parts was adopted as the modeling accuracy. We adopted the statistical indicators of the coefficient of determination (*R*^2^), the root-mean-square error (RMSE, μg/m^3^), the mean prediction error (MPE, μg/m^3^), and the relative prediction error (RPE, %) to evaluate the model performance. Meanwhile, the mapping effect of the PM_2.5_ spatial distribution as a feedback mechanism was also applied, to assist with the validation. The variables that caused obvious anomalies in mapping the continuous distribution of PM_2.5_ and that did not improve the estimation accuracy obviously were removed. Based on the multi-validation, the optimal combination of variables and the optimum network parameters could be selected.

## 4. Results and Discussion

Due to the cloud cover and bright surface with high reflectance [34], there are a lot of the missing data in the hourly AOT data for the central urban area of Wuhan during the research period. Therefore, we extracted two sample sets: (1) One set (Sample set A) containing approximately 80,000 sample pairs which are not matched with AOT; and (2) the other set (Sample set B) containing only about 1600 sample pairs that are matched with AOT.

### 4.1. Descriptive Statistics

**Figure 5:**
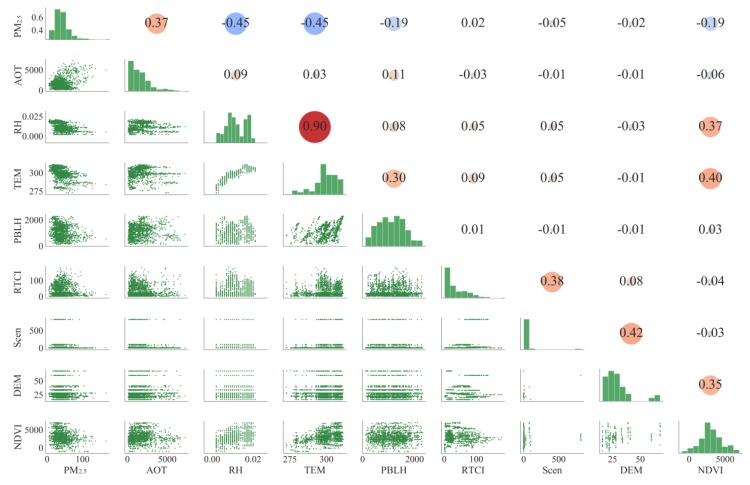
Diagram of the composite analysis matrix. The diagonal line of the matrix shows the histogram of the frequency distribution of each variable; the lower triangle shows the bivariate scatter plots; the upper triangle indicates the Pearson’s correlation coefficient between the two variables. Only parts of the variables are presented due to the page limit (composite analysis matrixes for the Sample set A and Sample set B are provided in Supplementary Material: Figures S1 and S2). “RTCI” means real-time check-in variable; “PBLH” means planetary boundary layer height; “TEM” means air temperature at a 2-m height; “RH” means specific relative humidity; “Scen” means the cleaner location type POI.

According to the statistics on data collected during the study period, the PM_2.5_ concentration, ranged from 2 μg/m^3^ to 209 μg/m^3^, with an average of 53.88 μg/m^3^ and a large variation on the spatiotemporal scale. Taking the Sample set B as an example shown in Figure 5. The diagonal line of the matrix visualizes the distribution of some variables. Excluding the NDVI, other variables are non-normally distributed (skewed distributed, multi-peak distributed, etc.). The bivariate scatter plots in the lower triangle show that PM_2.5_ exhibits a nonlinear relationship with most other variables. In the sample sets, the values of some variables are very unevenly distributed, which means that these features are less representative when training the model (Scen, DEM, etc.). From the upper triangle, we find that: (1) PM_2.5_ is negatively correlated with RH and TEM, which is exactly in line with a previous study in Wuhan [55]; (2) PBLH and NDVI also presents the negative relationships with PM_2.5_, in that a low atmospheric boundary layer height is not conducive to the diffusion and dilution of PM_2.5_, while vegetation can clean and purify the atmospheric environment; and (3) there are almost no linear correlations between social sensing variables and PM_2.5_. These results indicate that the traditional methods based on the assumption of linear relationships between variables would not be suitable to mine and explain these complex nonlinear relationships between variables.

### 4.2. Model Accuracy Evaluation

Both the model validation results and the mapping continuity of spatial distribution are important in applications. Sample set A for modeling, with an *R*^2^ of 0.832 (Table 1), obtained a higher accuracy than using Sample set B, which had an *R*^2^ of 0.742 (Table 2). And a more continuous spatiotemporal distribution could be obtained by using Sample set A. Accordingly, Sample set A was preferred for the modeling and evaluation. More details about the selection of sample sets are provided in the discussion in Section 4.4.

The effective and optimal variables appropriate for the DBN model were selected according to the modeling framework designed in this study and constrained during the entire study period. Finally, based on the experiments, a certain combination of variables (Time, NDVI, PM_s_, PM_t_, RTCI, TID, RH, TEM, EWS, NWS, SP, PBLH) resulted in the most optimized model accuracy, as well as the mapping results. Specially, the selection of social sensing variables is explained in more detail in the discussion in Section 4.3.

#### 4.2.1. Quantitative Evaluation Results

Table 1 lists the evaluation results generated by 10-fold CV. The model fitting *R*^2^ is 0.850, and the RMSE is 9.303 μg/m^3^. The CV results of *R*^2^, RMSE, MPE, and RPE are 0.832, 9.864 μg/m^3^, 6.961 μg/m^3^ and 23.764% respectively, which indicates that the model explains 83.2% of variability of the ground measured PM_2.5_. There is no over-fitting phenomenon by comparing the results of model fitting and CV, which indicates the reliability of the trained model.

For further exploration, we also investigated the effects of each kind of variable in the optimal variable combination A. As shown in Table 1, the accuracy of the model is reduced to varying degrees when any category of variables is removed. In particular, if the variables of RTCI and TID are removed, the *R*^2^ lowered 0.045 and the RMSE increased 1.22 μg/m^3^. Real-time check-in variable reflects the population activity and mobile patterns. Previous study found that particulate concentration hot spots are mainly distributed in urban centers where human activities accumulate, resulting in increased PM_2.5_ concentrations [39]. The automobile exhaust emission is one of the main sources of PM_2.5_ pollution in urban area [4]. Thus, both the RTCI and TID have great impacts on the model accuracy. As for NDVI, likely due to the potential filtering and absorption function of the vegetation [36], NDVI affected the model accuracy to a certain extent. The results demonstrated that with the integration of the remote sensing data NDVI, the dynamic social sensing data and other auxiliary data, the modeling result can be effectively promoted.

#### 4.2.2. Mapping Results of PM_2.5_ Concentration

**Figure 6:**
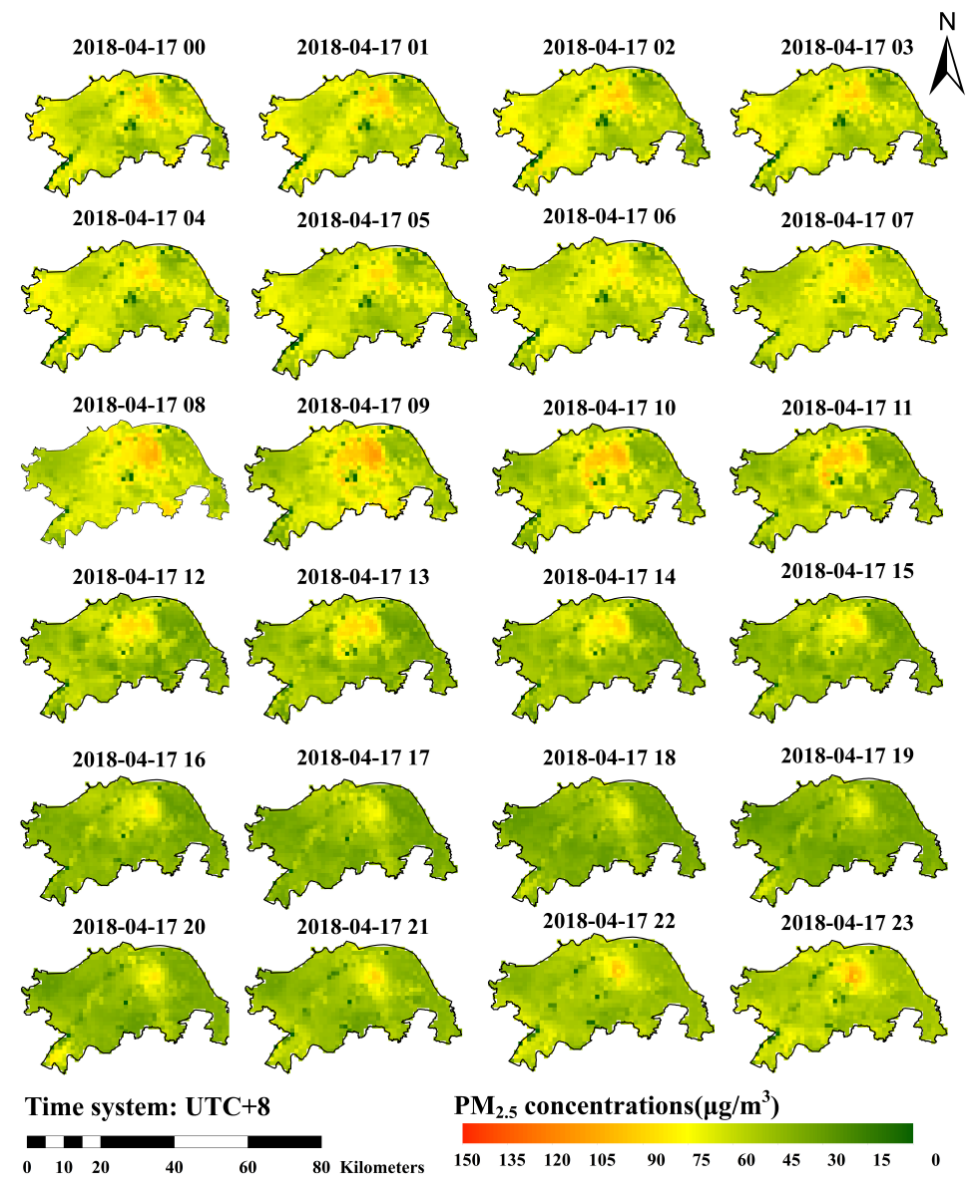
Spatial mapping of PM_2.5_ concentration over the 24 h of a day (time system: UTC + 8), with a spatial resolution of 0.01°.

Taking one day (17 April 2018) as an example, Figure 6 shows the 24 h spatial distribution of PM_2.5_ in the central urban area of Wuhan. Comparing with most studies using the AOT to predict PM_2.5_, we could also predict the PM_2.5_ distribution during the nighttime and obtain continuous mapping of hourly spatial distribution of PM_2.5_, since the input variables of the model were almost completely covered over time and space.

The mapping result reflects the spatiotemporal characteristics of PM_2.5_. Temporally, the hourly changes of PM_2.5_ concentration are obvious. Nevertheless, previous research has mostly considered daily, monthly, or even coarser temporal scales [10,15,16,18,19], which may hide the details of the hourly changes. The mapping results of this model provide more details of hourly PM_2.5_ concentration, which can be applied for urban real-time monitoring and air pollution prevention. With regard to the spatial scale, the precision of the 0.01° resolution can more precisely reflect the spatial details. The PM_2.5_ concentration in the Qingshan district, where heavy industries gather, shows a relatively high level, which is followed by the Hongshan district and the Wuchang district, where traffic and population is more concentrated. The literature on the spatiotemporal distribution of PM_2.5_ in Wuhan has also shown that the PM_2.5_ concentration in industrial areas, traffic areas and residential areas is higher than others [55]. What is more, the higher concentration spots in the low concentration areas can be identified as pollution emergencies.

**Figure 7:**
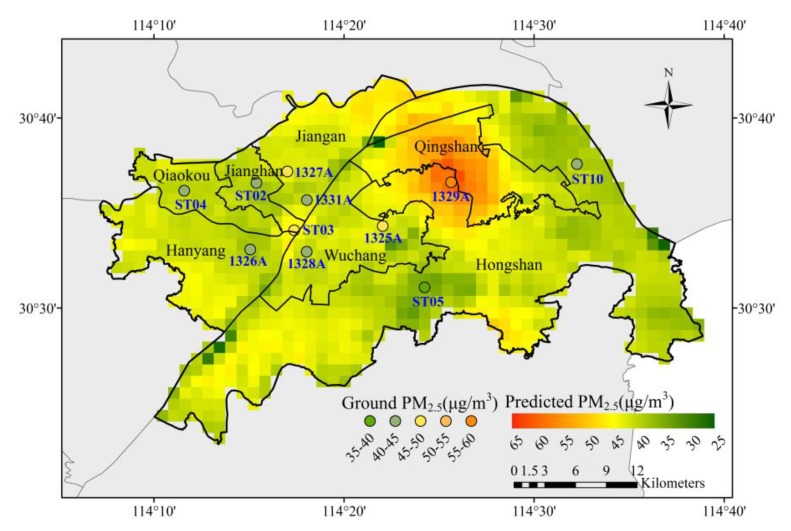
The spatial distribution of the average concentration of estimated PM_2.5_ and the average PM_2.5_ concentration for each monitoring station during the study period. (24 January 2018 to 31 July, 2018).

The PM_2.5_ distributions of larger temporal scales (daily, monthly, seasonal, etc.) can be generated by the hourly spatial mapping results. Figure 7 displays a map of the average concentration of estimated PM_2.5_ overlaid with the average concentration for each monitoring station during the study period. Satisfactorily, the two sets of data show good consistency. It can be seen that the spatial distribution of averaged PM_2.5_ concentration differ significantly in downtown Wuhan. PM_2.5_ pollution is serious in the Qingshan district, where station 1329A is located, since the Wuhan Iron and Steel Group Corporation is located in this area. The industrial waste gas emission has a bad effect on the air quality of the surrounding area. On the whole, most of the mapping grids of average concentration are higher than the annual average standard (35 μg/m^3^) set by the Air Quality Guidelines of China [56], and far from the standard set by the World Health Organization (10 μg/m^3^) [3].

### 4.3. The Effects of the Social Sensing Variables

**Figure 8:**
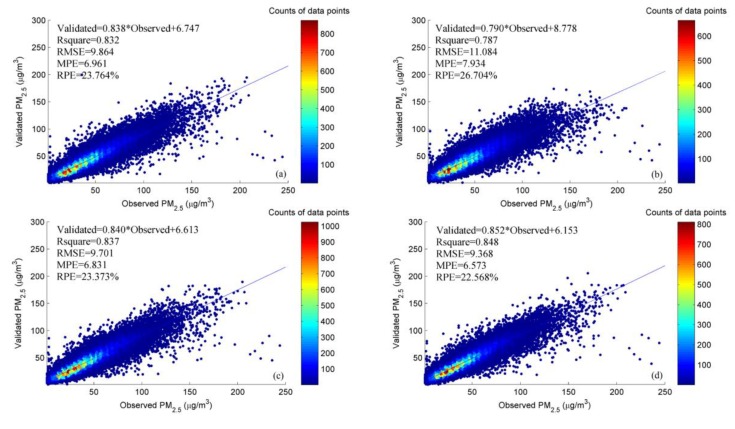
Scatter plots of the 10-fold CV results. (**a**) Optimal variable combination A (Time, NDVI, PM_s_, PM_t_, RTCI, TID, RH, TEM, EWS, NWS, SP, PBLH); (**b**) optimal variable combination A without RTCI and TID; (**c**) add ROAD into optimal variable combination A; (**d**) add POIs into optimal variable combination A. “ROAD” means the density of the road network.

**Figure 9:**
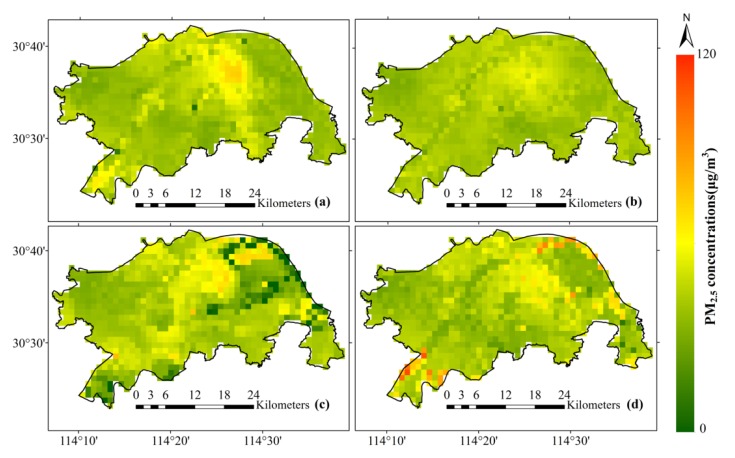
Mapping results of the estimated PM_2.5_ concentration using different variables. The variables used in (**a**–**d**) correspond to those in Figure 8 respectively.

In order to explore the effects of all kinds of social sensing variables, we conducted experiments on each social sensing variable and then verified the results. Figure 8 and Figure 9 show the quantitative and mapping results of the four models, respectively, corresponding to (a), (b), (c), and (d). As a contrast, (a) is the result using optimal variable combination A.

Analyzing the quantitative results, as shown in Figure 8b, without RTCI and TID in optimal variable combination A, *R*^2^ drops from 0.832 to 0.787, and the slope of the blue fit line (0.790) in Figure 8b is less than the slope (0.838) in Figure 8a. This means that RTCI and TID play positive effects in the model, in that they improve the model accuracy and reduce the extent of the underestimation. Figure 8c,d respectively show the validation results of adding ROAD and POIs (PS, Scen) into optimal variable combination A, where it can be seen that both variables can improve the model accuracy slightly (0.837, 0.848).

From the mapping results, Figure 9a presents the spatial distribution of PM_2.5_ for a given hour. The distribution of PM_2.5_ is generally consistent with the heterogeneity of the spatial distribution, and the transition is smooth. Figure 9b lacks more spatial details compared with Figure 9a, and the performance is insufficient in the high-value area. This illustrates that RTCI and TID are beneficial for the mapping of PM_2.5_ concentration. Figure 9c,d show that when adding ROAD and POIs to estimate PM_2.5_ concentration, the mapping results contain more outliers, and the spatial distribution of PM_2.5_ concentration also shows some differences with the results shown in Figure 9a. Obviously, we can see the low-value anomaly in the northeastern part of the downtown shown in Figure 9c, and the high-value anomaly in the southwestern part of the downtown shown in Figure 9d. This is probably because of the greater spatiotemporal heterogeneity of these relatively static variables. Furthermore, the representative samples from the monitoring stations in the study area are insufficient.

Taking both the quantitative evaluation and the mapping results into consideration, although ROAD and POIs can improve the estimation accuracy slightly, they bring obvious anomalies when mapping the continuous distribution of PM_2.5_. Thus, we finally removed these variables from our modeling process. Overall, the dynamic social sensing variables (RTCI, TID) that change in real time result a better performance than the relatively static data (POIs, ROAD) when the distribution of samples is sparse and heterogeneous.

### 4.4. The Dialectical Selection of the AOT Variable

Considering the wide used of AOT products in PM_2.5_ concentration estimation, we discuss the selection of the AOT variable in a practical scenario from two aspects. On the one hand, there are large gaps in the AOT data due to the restriction of conditions, as mentioned above. The intuitive performance is that the great quantity gap of the two sample sets (set A: 80,000 and set B: 1600) used for modeling. We compared the model results (Table 1 and Table 2) of the two sample sets and found that the validation result using sample set A for modeling obtains a higher accuracy, with an *R*^2^ of 0.832, than using sample set B, with an *R*^2^ of 0.742, which can be interpreted as the deep learning model needing a large amount of data to obtain more stable and accurate results. What is more, if we included AOT during the study period, there would be lots of gaps in the spatiotemporal mapping, and the coverage would be significantly reduced. Therefore, considering the limited temporal and spatial scope of the application scenarios, it is feasible to exclude the AOT variable, in order to obtain sufficient sample data and a more continuous mapping result.

On the other hand, the AOT variable can have a positive effect on the model accuracy. As shown in Table 2, which is based on Sample set B, *R*^2^ decreases by 0.033, when the AOT variable is excluded from the model. Overall, the dialectical selection of the AOT variable could be based on the practical application, considering the temporal and spatial conditions.

## 5. Conclusions

Mapping the hourly and continuously distributed PM_2.5_ is meaningful for air quality monitoring and health risk research. In this study, we mainly considered remote sensing data, social sensing data, meteorological data and the spatiotemporal features of PM_2.5_ to estimate hourly PM_2.5_ in the central area of Wuhan. A spatiotemporal grid matching framework was proposed to unify the multi-source heterogeneous data, and the DBN method was introduced to learn the complex relationships among variables. By exploring the effects of PM_2.5_ influencing factors in the estimation accuracy and mapping results of PM_2.5_ concentration, we came to the following conclusions: (1) The real-time check-in data and traffic index data have a positive influence on fine-scale air pollution studies, which dynamic characteristics can help to identify hot events; (2) when the relatively static variables vary widely in spatiotemporal scale and the representative samples are insufficient, these variables usually bring anomalies in the process of estimating PM_2.5_; (3) the AOT variable should be dialectically selected, such as considering the limited modeling conditions, whether the PM_2.5_ concentration at night is needed and whether the full-coverage mapping results can be obtained.

Further study will focus on improving the model ability to learn rare features, and we will look to explore the intensive observations data for monitoring the air pollutants [57,58]. It is worthwhile to integrate remote sensing and social sensing for spatiotemporal estimation of air pollution based on advanced statistical methods. We hope that this paper will inspire researchers to study the integration of multi-source data using advanced methods for urban ecological applications.

## Figures and Tables

**Table 1:** Model validation using Sample set A.

Variables	Model Fitting				10 Fold Cross-Validation			
	*R* ^2^	RMSE ^1^	MPE ^2^	RPE ^3^ (%)	*R* ^2^	RMSE	MPE	RPE (%)
optimal variables A ^4^	0.850	9.303	6.683	22.412	0.832	9.864	6.961	23.764
without RTCI ^5^, TID ^6^	0.792	10.966	7.889	26.418	0.787	11.084	7.934	26.704
without PM_s_^7^, PM_t_^8^	0.830	9.916	7.180	23.890	0.810	10.478	7.573	25.244
without Wea ^9^	0.831	9.888	7.021	23.822	0.824	10.092	7.099	24.313
without NDVI	0.833	9.813	7.057	23.643	0.810	10.467	7.414	25.216
without Time	0.825	10.065	7.158	24.250	0.821	10.168	7.198	24.496

^1^ “RMSE” means the root-mean-square error; ^2^ “MPE” means the mean prediction error; ^3^ “RPE” mans the relative prediction error; ^4^ “optimal variables A” means the optimal variable combination based the Sample set A (Time, NDVI, PM_s_, PM_t_, RTCI, TID, RH, TEM, EWS, NWS, SP, PBLH); ^5^ “RTCI” means real-time check-in variable; ^6^ ”TID” means traffic index variable; ^7^ “PM_s_” means the spatial feature of PM_2.5_; ^8^ “PM_t_” means the temporal feature of PM_2.5_; ^9^ “Wea” means the meteorological variables.

**Table 2:** Model validation using Sample set B.

Model	Model Fitting				10 Fold Cross-Validation			
	*R* ^2^	RMSE	MPE	RPE (%)	*R* ^2^	RMSE	MPE	RPE (%)
optimal variables B ^1^	0.834	8.136	6.152	19.647	0.742	10.161	7.478	24.537
without AOT	0.798	9.001	6.836	21.737	0.709	10.821	7.965	26.131

^1^ “optimal variables B” means the optimal combination of variables based on Sample set B (AOT, Time, NDVI, PM_s_, PM_t_, RTCI, TID, RH, TEM, EWS, NWS, SP, PBLH).

## Supplementary Materials

The following are available online at https://www.mdpi.com/1660-4601/16/21/4102/s1, Table S1: Abbreviations for data sets and variables, Figure S1: Diagram of the composite analysis matrix based on Sample set A, Figure S2: Diagram of the composite analysis matrix based on Sample set B.
