# Supplementary material for: Integration of Remote Sensing and Social Sensing Data in a Deep Learning Framework for Hourly Urban PM2.5 Mapping
Source: Int J Environ Res Public Health. 2019 Oct 24;16(21):4102. doi: 10.3390/ijerph16214102 (PMC6861963; doi:10.3390/ijerph16214102)
Supplement: Supplementary file 1 [file ijerph-16-04102-s001.pdf]

Supplementary Materials

Table of Contents

1. Abbreviations for data sets and variables **(Table S1)**
2. Composite analysis matrixes based on the Sample set A and Sample set B **(Figure S1, Figure S2)**

1. Abbreviation for data sets and variables.

Table S1: Abbreviations for data sets and variables.

Date set	Variable		Abbreviation	
Site $PM_{2.5}$	$PM_{2.5}$		$PM_{2.5}$	
	spatial feature of $PM_{2.5}$		PM_s	
	temporal feature of $PM_{2.5}$		PM_t	
Social sensing data(SSD)	real-time check-in		RTCI	
	traffic index density		TID	
	road network density		ROAD	
	point of interests	potential sources of pollution type	POIs	PS
		cleaner location type		Scen
Remote sensing data(RSD)	aerosol optical thickness		AOT	
	normalized difference vegetation index		NDVI	
Meteorological data(Wea)	relative humidity		RH	
	air temperature		TEM	
	east wind speed, north wind speed		EWS, NWS	
	surface pressure		SP	
	planetary boundary layer height		PBLH	
Terrian data	digital elevation model product		DEM	

2. Composite analysis matrixes based on the Sample set A and Sample set B.

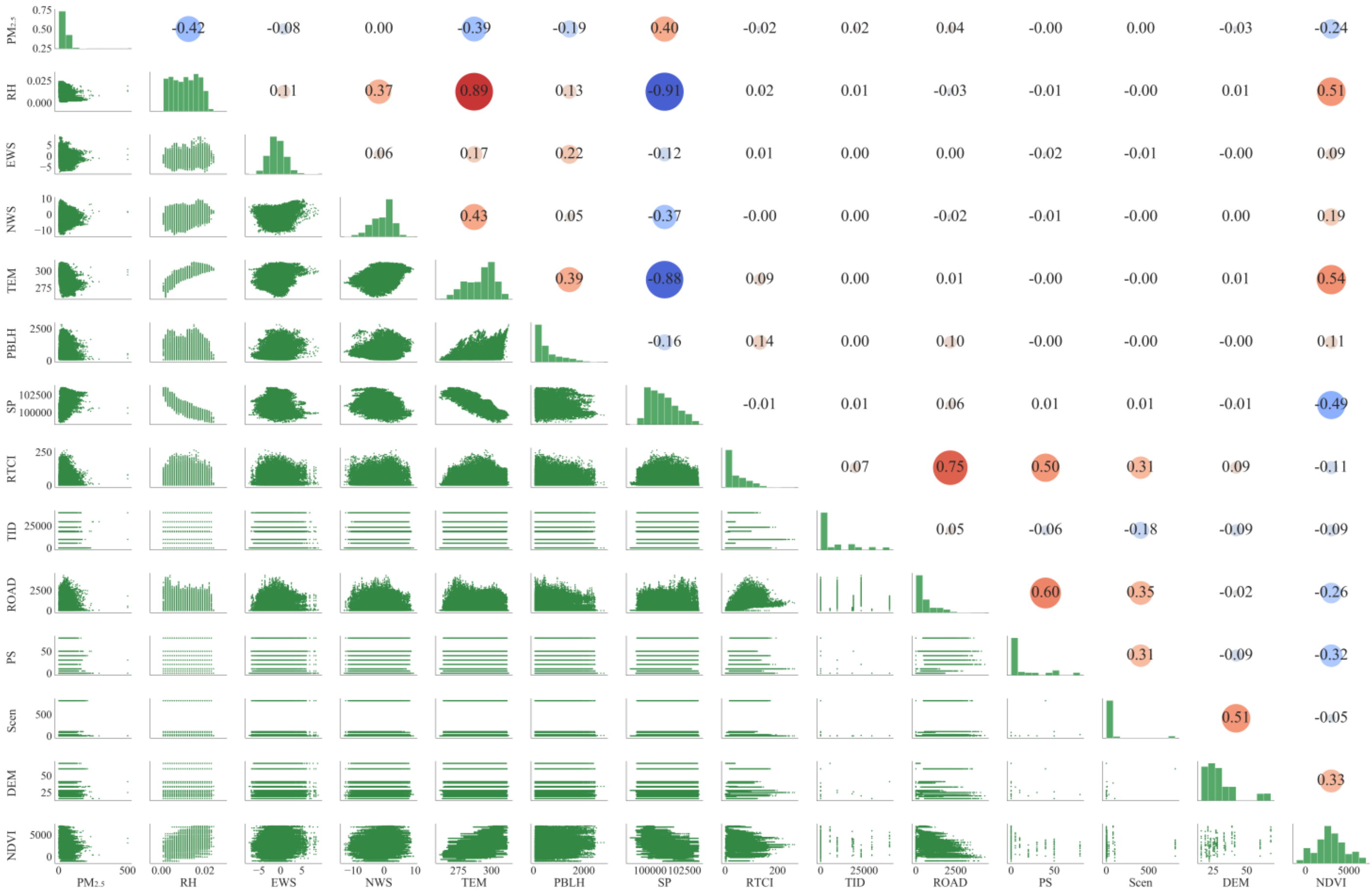

Figure S1: Diagram of the composite analysis matrix based on Sample set A.

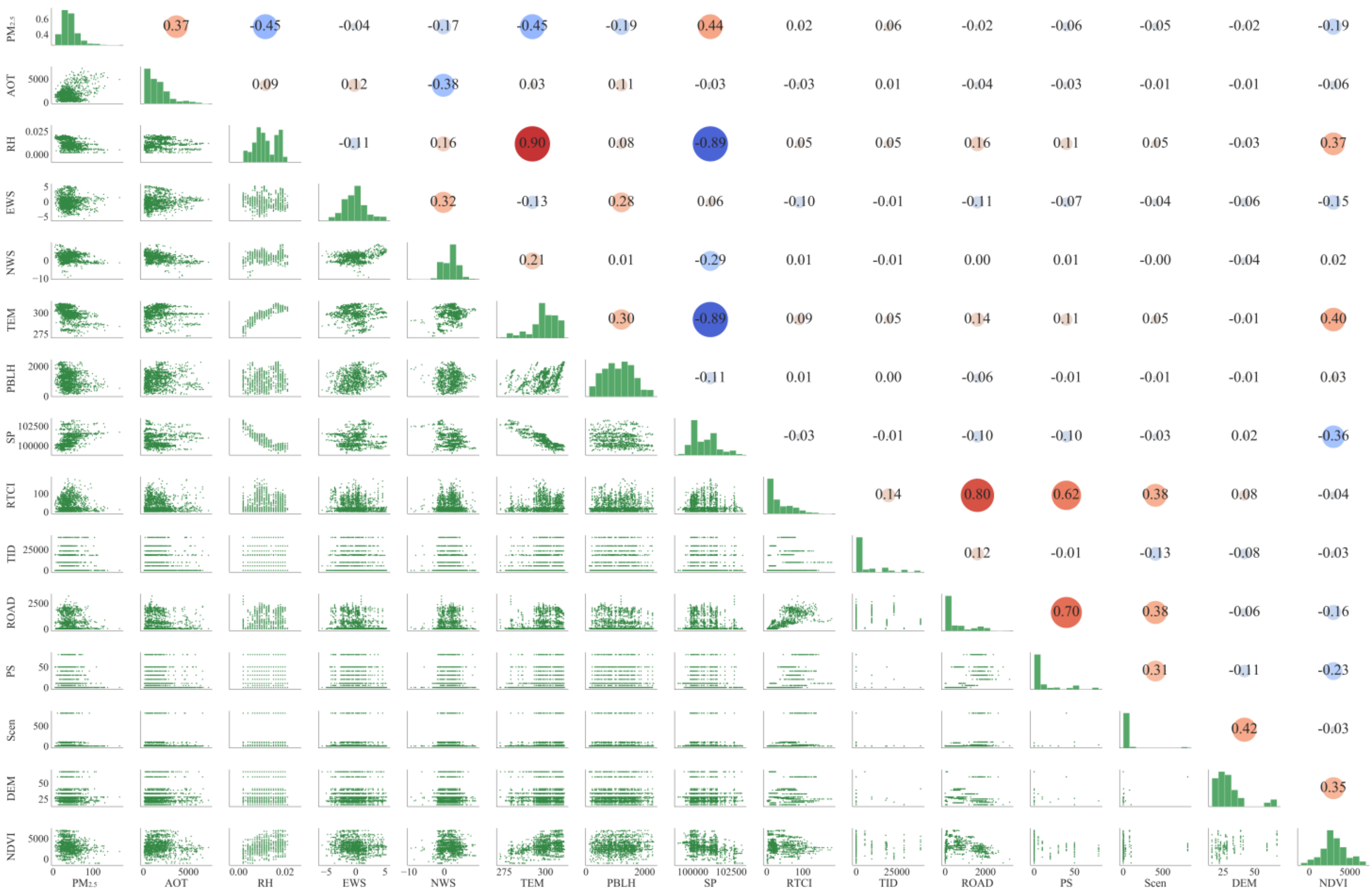

Figure S2: Diagram of the composite analysis matrix based on Sample set B.
